# Light-Activatable Transfection System Using Hybrid Vectors Composed of Thermosensitive Dendron Lipids and Gold Nanorods

**DOI:** 10.3390/pharmaceutics12030239

**Published:** 2020-03-07

**Authors:** Takuya Hashimoto, Tomoya Hirata, Eiji Yuba, Atsushi Harada, Kenji Kono

**Affiliations:** Department of Applied Chemistry, Graduate School of Engineering, Osaka Prefecture University, 1–1 Gakuen–cho, Naka–ku, Sakai, Osaka 5998531, Japan; sv108049@edu.osakafu-u.ac.jp (T.H.); sdhrii47ciodjspytisqi6vnxis@gmail.com (T.H.); biopolymer@chem.osakafu-u.ac.jp (K.K.)

**Keywords:** gene delivery, near infrared light, gold nanorod, dendron, thermosensitivity, endosome escape

## Abstract

Background: Gene delivery to target cells is crucially important to establish gene therapy and regenerative medicine. Although various virus-based and synthetic molecule-based gene vectors have been developed to date, selective transfection in a site or a cell level is still challenging. For this study, both light-responsive and temperature-responsive synthetic gene vectors were designed for spatiotemporal control of a transfection system. Methods: 11-Mercaptoundecanoic acid-coated gold nanorods were mixed with polyamidoamine dendron-bearing lipids of two types having amino-terminus or ethoxydiethylene glycol-terminus to obtain hybrid vectors. Hybrid vectors were mixed further with pDNA. Then we investigated their physicochemical properties and transfection efficacy with or without near infrared laser irradiation. Results: Hybrid vectors formed complexes with pDNA and exhibited enhanced photothermal property under near infrared laser irradiation compared with parent gold nanorods. Transfection efficacy of complexes was promoted considerably by brief laser irradiation soon after complex application to the cells. Analysis of intracellular distribution revealed that laser irradiation promoted the adsorption of complexes to the cells and cytosolic release of pDNA, which is derived from the change in surface hydrophobicity of complexes through dehydration of temperature-responsive groups. Conclusions: Hybrid vector is promising as a light-activatable transfection system.

## 1. Introduction

Introduction of nucleic acids into target cells is a basic biological technology. In addition, efficient nucleic acid delivery technology is desired to establish novel therapeutic approaches related to gene therapy and regenerative medicine. Virus-based gene vectors have effective gene transduction capability because they can use intrinsic infection mechanisms of viruses to the cells. However, immunogenicity and safety issues on virus-based gene vectors remain controversial [1,2]. Instead of virus-based systems, synthetic molecules such as cationic peptides, lipids, and polymers have been developed for application to gene delivery carriers [3,4,5]. Gene transduction efficacy of these synthetic gene vectors is low compared with that of virus-based systems. Furthermore, selectivity of gene delivery using synthetic vectors at a site, tissue, and cell level with desired timing and duration remains challenging.

One potential strategy to achieve such selective gene transfection is the utilization of stimulus-responsiveness. Gene vectors with stimulus-responsivity are expected to induce selective association to a target site/cell, internalization to the cells, and endosomal escape of vectors in response to application of external stimuli, which engenders “on-demand” transfection. A representative stimulus used for control of gene transfection is temperature. Temperature can be handled easily and can be used safely. Typically, poly(*N*-isopropylacrylamide)-based pDNA polyplexes have been used for control of interaction with cells via temperature [6,7].

Light-controlled gene delivery system is another candidate for stimulus-responsive selective gene transfection. Light is useful because of its spatiotemporal controllability. Especially, near-infrared (NIR) light penetrates biological tissues and skin with sufficient depth because light absorption of water molecules and endogenous chromophores such as hemoglobin, oxyhemoglobin and melanin is low [8,9,10]. NIR light-responsive gene delivery carriers have been studied intensively using nanomaterials that have strong absorption at the NIR region [11]. Among these nanomaterials, gold nanoparticles including nanostars, nanorods, nanoshells, and nanocages have gained much attention because of their photothermal properties derived from localized surface plasmon resonance (LSPR) [12,13]. Gold nanorod (AuNR) has a high absorption coefficient at LSPR peak, compared with to those of gold nanospheres and gold nanoshells [14]. Furthermore, spectra including LSPR peak of AuNR can be changed from visible to the NIR region by adjusting the aspect ratio of AuNR [15,16]. Considering such unique characteristics of AuNR, cationic polymer-modified AuNRs have been developed to provide the complex formation property with nucleic acids and photo-responsive release systems of nucleic acid using near infrared (NIR) light [17,18]. Nevertheless, no report has described a study of both temperature-responsive and light-responsive AuNR-based gene delivery carriers.

We earlier developed synthetic gene vectors using polyamidoamine (PAMAM) dendron-bearing lipids that are fusion molecules of PAMAM dendrimer and cationic lipids [19,20,21]. Dendron lipids can form complex with plasmid DNA (pDNA) via electrostatic interaction derived from terminal amino groups in dendron moiety. After internalization to the cells, dendron lipids promote endosome escape of pDNA through membrane fusogenic activity derived from alkyl chains and pH-buffering effects at weakly acidic environments by tertiary amines in PAMAM dendrons, leading to efficient transfection [19,20]. Terminal amino groups can be functionalized to introduce desired properties to dendron lipid-based gene carriers such as colloidal stability and targeting properties by the respective modification of poly (ethylene glycol) and galactose [21,22]. We also developed temperature-responsive dendron lipids by introducing side chain structures of temperature-responsive polymers [23,24,25]. Isobutyramide (IBAM) group-modified dendron lipid-based self-assemblies showed drastic morphology change in response to a temperature increase that is derived from change in the molecular shape after dehydration of IBAM moiety at high temperature [23,24]. Introduction of oligo(ethylene glycol) groups to dendron lipids also provided temperature-sensitive vesicles that can promote adsorption and internalization to cells via hydrophobic interactions at higher temperatures [25].

**Figure 1:**
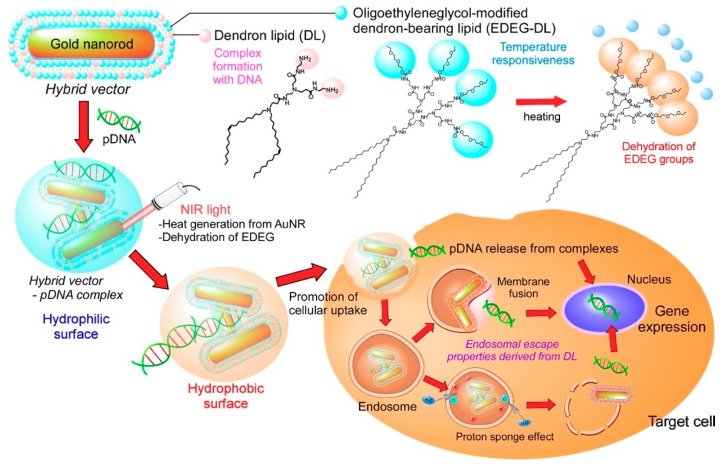
Design of light-activatable transfection system using complexes of pDNA with hybrid vectors composed of gold nanorods (AuNRs) covered with amino group-terminated dendron-bearing lipids (DL) and oligo (ethylene glycol) group-terminated dendron-bearing lipids (EDEG-DL). Amino groups of DL interact with pDNA via electrostatic interaction to form submicrometer-sized complexes. Ethoxydiethylene glycol group-introduced PAMAM G2 dendron-bearing lipid (EDEG-DL) possesses temperature-sensitivity that changes its surface property from hydrophilic to hydrophobic at high temperature conditions. AuNR absorbs near infrared (NIR) light and converts light energy to heat energy. Generated heat changes the surface properties of complexes, which promotes cellular association of complexes via hydrophobic interaction and release of pDNA into cytoplasm to enhance gene expression only at NIR light irradiated cells or tissues.

Considering these unique characteristics of dendron lipids, we designed both light-responsive and temperature-responsive gene vectors to produce a light-activatable transfection system (Figure 1). The surface of AuNR was covered with dendron lipids of two types: PAMAM G1 dendron-bearing lipid with two oleyl chains (DL) for complex formation with pDNA via amino groups in dendron moiety and ethoxydiethylene glycol group-introduced PAMAM G2 dendron-bearing lipid (EDEG-DL) to provide temperature-responsiveness to the vectors. These surfaces covered with AuNRs were designated as a hybrid vector. The cellular association of pDNA-hybrid vector complexes would be suppressed because the complex surface is covered with hydrated EDEG groups at a physiological temperature. When an NIR laser is irradiated to the complex, AuNR generates heat, which induces the dehydration of EDEG groups and the change of complex surface properties from hydrophilic to hydrophobic, leading to the internalization to the cells. Generated heat under NIR light irradiation would also promote the liberation of pDNA from the complex, which can achieve gene transduction. Here, the preparation of hybrid vector composed of AuNR and functional dendron lipids, the characterization of pDNA-hybrid vector complexes and their application to light activatable transfection system were demonstrated.

## 2. Materials and Methods

### 2.1. Materials

PAMAM G1 dendron-bearing lipid having two oleyl groups (DL) was synthesized as previously described [26]. Ethoxydiethylene glycol (EDEG)-modification to PAMAM G2 dendron-bearing lipid having two octadecyl groups (DL−S−G2) was performed by the reaction of amino groups of DL−S−G2 and EDEG-4-nitrophenyl chloroformate according to previous reports [27] (See also Scheme S1 in Supplementary Materials). Synthesized EDEG−DL was characterized using NMR. Hoechst33342 and LysoTracker Green DND-26 were obtained from Invitrogen (Eugene, OR, USA). Lissamine rhodamine B-sulfonyl phosphatidylethanolamine (Rh−PE) were purchased from Avanti Polar Lipids (Birmingham, AL, USA). Agarose were obtained from Nacalai Tesque (Kyoto, Japan). Plasmid DNA (pDNA) encoding pEGFP−C1 was obtained from Clontech (Mountain View, CA, USA). pDNA encoding firefly luciferase (pGL4−Luc) was obtained from Promega (Madison, WI, USA). pDNA were purified using a FastGene Plasmid Mini Kit from Nippon Genetics (Tokyo, Japan). Rhodamine-labeled pGL4-Luc was prepared by using Label IT^®^CX-Rhodamine Labeling Kit from Takara Bio Inc. (Shiga, Japan). Fetal bovine serum (FBS) was obtained from MP biomedical, Inc. (Irvine, CA, USA). Dulbecco’s modified Eagle’s medium (DMEM) was purchased from Nissui Pharmaceutical (Tokyo, Japan).

### 2.2. Preparation of MUA-AuNR

Surface ligand exchange of 11-mercaptoundecanoic acid (MUA) on CTAB-AuNRs was conducted according to previous literature [28,29]. Briefly, CTAB-coated AuNR was synthesized using a one-step seedless growth method [28], and the excess CTAB was removed by repeated centrifugation (15,000 rpm, 40 °C, 30 min, 3 times). 0.2602 g of MUA dissolved in 60 mL of 0.05 M NaOH aq. was added into CTAB-AuNR aqueous suspension (0.5 mM, 150 mL). The mixture was vigorously stirred at room temperature for 24 h, then centrifuged at 8,000 rpm for 20 min. After adjusting the solution pH to 12 using NaOH aq., the excess reagents were removed by repeated centrifugation (8,000 rpm, 30 °C, 20 min, 2 times). The resultant MHA-AuNR was redispersed in aqueous solution at pH 11.5 (Au concentration: 3.32 ± 0.73 mM).

### 2.3. Preparation of Hybrid Vector and Complex

To a dry thin membrane of the EDEG−DL/DL (9/1, mol/mol), a given volume of 10 mM phosphate buffer (pH 3.0) was added and sonicated for 5 min using a bath type sonicator to prepare a lipid suspension (1.64 mM, DL suspension). Subsequantly, MUA−AuNR dispersion was added to lipid suspension (Au/lipid = 9/10, mol/mol) and solution pH was adjusted to pH 7.4 by adding NaOH aquoeous solution and sonicated for 5 min (hybrid vector). pDNA (1 µg) dissolved in 20 mM Tris−HCl buffer (pH 7.4, 50 µL) was added to a given amount of hybrid vector suspension and incubatated for 30 min at room temperature to obtain complexes with varying ratios of DL lipids to pDNA phosphate (L/P ratio).

### 2.4. General Characterization

Au concentration of each sample was determined using SPS7800 ICP-MS spectrometer (Seiko Instruments Inc., Chiba, Japan). Morphology of DL suspension, MUA−AuNR, hybrid vector and complex were analyzed using atomic force microscopy (AFM) using a probe station and a unit system of the scanning probe microscopy system (SPI3800, SPA400; Seiko Instruments Inc., Chiba, Japan). The silicon cantilever (SI-DF40; Seiko Instruments Inc., Chiba, Japan) had a spring constant of 16 N/m. Each sample suspension was applied to freshly cleaved mica and incubated on the mica for 30 min. Measurements were taken in dynamic force mode (noncontact mode). Morphology of MUA−AuNR, hybrid vector and complex were further analyzed using transmission electron microscopy (TEM) (JEOL Ltd., JEM-2000FEX II, Tokyo, Japan) operated at 200 kV. Before TEM observation, the dispersion was dropped on a carbon-coated copper grid, and then excess of dispersion was removed with a filter paper. Then 10 µL of 2% aqueous sodium phosphotungstate solution was dropped on a grid and dried in a desiccator overnight.

The absorption spectra for AuNR, hybrid vector and complexes were analyzed using a Jasco V-670 spectrophotometer (Jasco Inc., Tokyo, Japan) at a wavelength range of 400–1100 nm. For analysis of photothermal property, MUA−AuNR, hybrid vector or complex (Au concentration: 13.5 µg/mL, 1 mL) in DMEM suppremented with 10% FBS without phenol red was placed in a quartz cell. NIR laser (YHTC Co., Ltd, KLD-3ALT, λ = 808 nm, 3.5 W/cm^2^, Tokyo, Japan) was irradiated to the solution and the change in temperature of the solution was monitored using a thermometer (SK-1250MC, SATO KEIRYOKI MFG. Co., Ltd., Tokyo, Japan). Initial temperature of the dispersion was fixed to be 25 ± 0.2 °C. Transmittance of MUA−AuNR, hybrid vector, complex suspension (lipid concentration: 0.1 mg/mL, 2 mL) in 10 mM phosphate and 140 mM NaCl solution at pH 7.4 at 660 nm was measured using a Jasco V-670 spectrophotometer equipped with a Peltier type thermostatic cell holder coupled with a controller ETC-505T. Heat rate of sample cells was adjusted to 2.0 °C·min^−1^.

Diameters and zeta pontentials of DL suspension, MUA−AuNR, hybrid vector and complex in 0.1 mM phosphate aqueous solution at 25 °C or 50 °C were measured using a Zetasizer Nano ZS (Malvern Instruments Ltd., Worcester-shire, UK).

### 2.5. Agarose Gel Electrophoresis

Hybrid vector−pDNA complexes with varying L/P ratios were prepared by mixing pDNA (0.15 μg) dissolved in 20 mM Tris-HCl buffer (5 μL) and hybrid vector suspension (5 μL). After 30 min incubation at room temperature, the samples (10 μL) were electrophoresed on 0.6 wt% agarose gel in 40 mM Tris, 20 mM sodium acetate, and 2 mM EDTA buffer (pH 8.0) containing 1 μg/mL ethidium bromide at 100 V for 30 min. The ethidium bromide-stained bands were visualized using a LAS-1000 UV mini (Fujifilm, Tokyo, Japan) and analyzed with Science Lab 2003 Multi Gauge software (Fujifilm, Tokyo, Japan). To examine the influence of NIR light irradiation on pDNA release from hybrid vector, the mixture of hybrid vector dispersion and pDNA after the 30 min-incubation was suspended in a quartz cell (Au concentration: 13.5 µg/mL, 0.1 mL) at 37 °C and NIR laser (λ = 808 nm, 3.5 W/cm^2^) was irradiated to dispersion for 1 min. Then, the aliquots (10 µL) of dispersion were electrophoresed and the ethidium bromide-stained bands were visualized in the same way as the above.

### 2.6. Transfection

Human cervix adenocarcinoma-derived HeLa cells obtained from Cell Resource Center for Biomedical Research, Tohoku University (Sendai, Japan) were grown in DMEM supplemented with 10% FBS and antibiotics at 37 °C under 5% CO_2_. HeLa cells were seeded into a 96-well microplate (1 × 10^4^ cells/well) in 100 µL of DMEM supplemented with 10% FBS and cultured overnight. 10 µL of aliquots of complex dispersion containing pEGFP−C1 was added to the cells (0.05 µg pDNA/well) and incubated at 37 °C. As depicted in Figure 3, NIR laser (λ = 808 nm, 3.5 W/cm^2^) was irradiated for given times and then incubated with complexes for total 24 h. Under the condition without laser irradiation, complex was incubated for 24 h. The cells were washed three times with PBS containing 0.36 mM CaCl_2_ and 0.42 mM MgCl_2_ (PBS(+)), and cultured for additional 24 h. Then, detached cells using 0.25% trypsin were applied to flow cytometric analysis (CytoFlex, Beckman Coulter, Inc., Brea, CA, USA) to evaluate GFP-expressing cells.

### 2.7. Cellular Association of Complexes

For analysis of cellular association of complexes or pDNA with cells, DL suspension containing 0.6 mol% Rh-PE as a lipid component or Rh-labeled pDNA were used for preparation of complexes, respectively. HeLa cells (1 × 10^5^ cells) cultured overnight in a 96-well plate were washed with PBS(+) and then incubated in culture medium. Fluorescently labeled complexes were added gently to the cells and incubated for 24 h at 37 °C. The cells were washed with PBS(+) three times, and then the detached cells using 0.25% trypsin were applied to flow cytometry. For light irradiation, NIR laser (λ = 808 nm, 3.5 W/cm^2^) was irradiated for 1 min at 1 h after complex apply to the cells.

### 2.8. Intracellular Distribution Analysis

HeLa cells (2 × 10^5^ cells) were seeded on a 35 mm glass-bottom dish in 2 mL of DMEM supplemented with 10% FBS and cultured overnight. After cells were washed with PBS three times, fluorescently labeled complexes were added gently to the cells. After 1 h-incubation, NIR laser (λ = 808 nm, 3.5 W/cm^2^) was irradiated for 1 min to the cells and then incubated further 0.5 or 23 h. Under the condition without laser irradiation, complex was incubated for 1.5 or 24 h. After cells were gently washed with PBS twice, intracellular organelle was stained with LysoTracker Green DND-26 and Hoechst33342 according to the manufacturer’s instructions. Confocal laser scanning microscopic (CLSM) analysis of these cells was performed using LSM 5 EXCITER (Carl Zeiss Co., Ltd., Oberkochen, Germany).

### 2.9. Statistical Analysis

Statistically significant differences between experimental groups were determined using Prism software (v8, GraphPad). Where one-way ANOVA followed by Tukey’s HSD post hoc test was used, variance between groups was found to be similar by Brown-Forsythe test. For single comparisons, a two-tailed Student’s *t*-test was used.

## 3. Results

### 3.1. Characterization of Hybrid Vectors and Complexes

**Figure 2:**
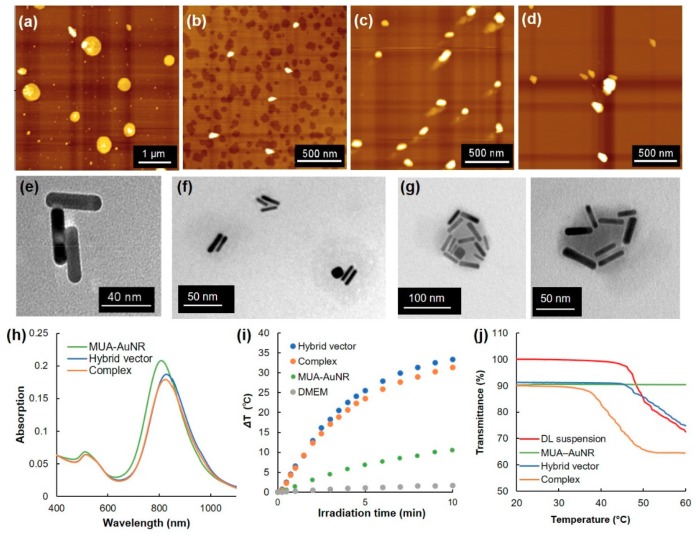
Atomic force microscopic (AFM) images for (**a**) dendron lipid suspension (EDEG-DL/DL = 9/1, mol/mol), (**b**) MUA-AuNR, (**c**) hybrid vector (Au/lipid = 9/10, mol/mol) and (**d**) complex (L/P = 48). Transmission electron microscopic (TEM) images for (**e**) MUA-AuNR, (**f**) hybrid vector, and (**g**) complex. (**h**) Absorption spectra for MUA-AuNR, hybrid vector and complex. (**i**) Heat generation of DMEM suspension for MUA-AuNR, hybrid vector and complex under NIR laser irradiation as a function of the laser irradiation time. (**j**) Transmittance for dendron lipid suspension, MUA-AuNR, hybrid vector and complex as a function of temperature.

Thermosensitive dendron lipid (EDEG-DL) was synthesized as reported earlier [25] (Scheme S1). A mixed lipid suspension of EDEG-DL and DL (9/1, mol/mol) formed spherical particles with heterogeneous size distribution and positively charged zeta potential (Figure 2a and Table 1). Also, MUA-coated AuNR was prepared by surface ligand exchange of CTAB-coated AuNRs using MUA. Analyses by AFM, TEM, and DLS revealed that MUA-AuNR with aspect ratios of 4, 40 nm longitudinal size, and negatively charged zeta potential were obtained (Figure 2b,e, Figure S1 and Table 1). After mixing DL suspension and MUA-AuNR, ellipsoidal particles of approximately 100 nm size were observed from AFM analysis (Figure 2c). According to the TEM image, several mutually associated AuNRs and slightly shaded spherical regions were observed around associated AuNRs (Figure 2f and Figure S1). DLS data showed size of 130 nm (Table 1), which is apparently a larger size than MUA-AuNR and smaller than DL suspension, suggesting the formation of hybrid vector composed of several AuNRs covered with DL lipids.

Because the prepared hybrid vector possesses slightly positively charged zeta potential (Table 1), amino groups derived from DL might be exposed on the surface of hybrid vectors, which is useful for complex formation with pDNA via electrostatic interaction. pDNA and hybrid vectors were mixed at various lipid/DNA ratios (L/P ratios) and agarose gel electrophoretic analysis was employed to confirm the complex formation (Figure S2). With increasing L/P ratio, free pDNA bands decreased (Figure S2a). At L/P = 48, retardation of DNA band was inhibited completely, indicating the completion of complex formation between pDNA and hybrid vectors. From AFM images and DLS analysis, slightly large particles (around 200 nm) than those of the parent hybrid vector (130 nm) were observed (Figure 2d and Table 1). Furthermore, TEM images for complexes revealed that many AuNRs were associated into a single particle with a shaded region (Figure 2g and Figure S1). Simultaneously, particles with one or two AuNRs were observed, which might be hybrid vectors that did not associate to the complexes (Figure S1).

Optical properties of AuNRs during complex formation were analyzed (Figure 2h). MUA−AuNR showed transverse and longitudinal peaks respectively near 520 nm and 820 nm. Hybrid vector and the complex with pDNA also showed almost identical absorption spectra with parent MUA-AuNR. Considering that hybrid vector and complex have strong absorption at NIR region, photothermal properties under NIR laser irradiation were investigated (Figure 2i). Surprisingly, the hybrid vector and complex exhibited significantly enhanced heat generation behaviors under NIR laser irradiation compared with parent MUA-AuNR, even though the Au concentration was equal for each suspension. Such rapid heat generation would be useful for photothermal control of thermosensitive materials. Therefore, the temperature-sensitive property of the complex was evaluated by measuring the transmittance change of each sample (Figure 2j). The MUA-AuNR suspension showed no transmittance change under the experimental temperature condition. Significant transmittance decrease for DL suspension was observed at around 50 °C, suggesting the aggregation of particles because of surface property changes through dehydration of EDEG moieties. This phenomenon was also confirmed from DLS results by the size increase at 50 °C (Table 1). Hybrid vector also showed almost identical behavior to that of DL suspension (Figure 2j and Table 1), whereas complexation with pDNA decreased transmittance at approximately 40 °C.

### 3.2. Transfection

Considering the light-responsive and temperature-responsive properties of complex, these complexes were applied to a light-activatable transection system. First, L/P ratio was optimized for transfection of pEGFP-C1 DNA (Figure S3). With increase of the L/P ratio, GFP fluorescence from transfected cells gradually increased (Figure S3a), which might correspond to increased cellular association of complexes (Figure S3b). To optimize the laser irradiation time, cells were irradiated with an NIR laser at 1 h after apply of complex at L/P 48 (Figure S4). GFP expression levels and GFP-positive cells increased considerably when the NIR laser was irradiated for 1 min, whereas further laser irradiation showed almost identical transfection activity with cells transfected without laser irradiation. Therefore, L/P 48 and 1 min irradiation were used for the following experiments.

**Figure 3:**
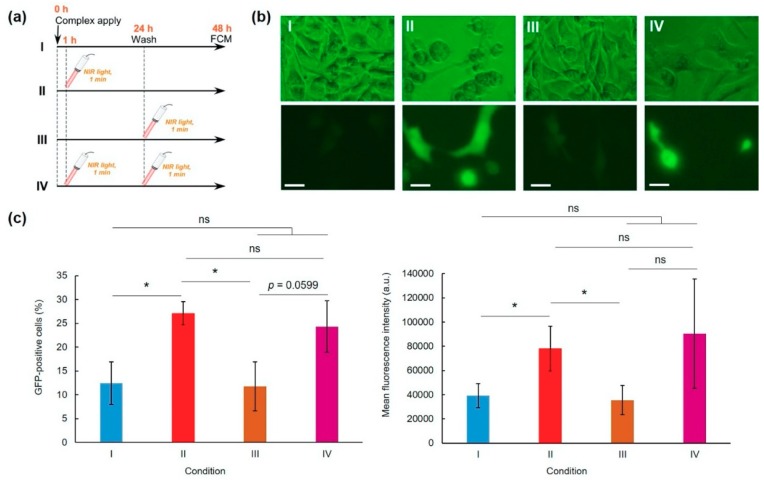
Effects of NIR laser irradiation condition on pDNA-hybrid vector complex transfection activity (L/P = 48). (**a**) Experimental conditions. HeLa cells were incubated with complexes for 24 h and were washed three times using PBS. After additional 24 h culture, GFP expression was evaluated using a flow cytometer: condition I, no laser irradiation; condition II, NIR laser irradiation at 1 h after sample apply; condition III, NIR laser irradiation at 24 h after sample apply; and condition IV, NIR laser irradiation applied at 1 and 24 h after sampling. Representative fluorescence images (**b**) and GFP-positive cells and GFP expression levels (**c**) of HeLa cells under various conditions (*n* = 3–4). Scale bars represent 10 µm. Statistical analyses were done using analysis of variance (ANOVA) with Tukey’s test. * *p* < 0.05 compared with other groups, ns; not significant.

Next, the laser irradiation timing effects on transfection activity were evaluated (Figure 3 and Figure S5). Compared with a no laser irradiation condition (Condition I), laser irradiation at 1 h (Condition II) significantly enhanced the GFP expression (Figure 3b,c). However, laser irradiation at 24 h (Condition III) showed no transfection activity enhancement. Double irradiation at 1 and 24 h (Condition IV) also increased average transfection activity, but not significantly more than for the no irradiated group (Figure 3c). Therefore, single and brief laser irradiation applied soon after sample apply are suitable to improve and control transfection activity.

### 3.3. Intracellular Behavior

**Figure 4:**
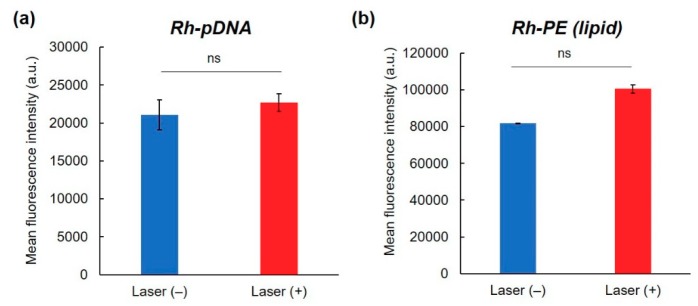
Effect of NIR laser irradiation on cellular association of complexes. Fluorescence intensity of HeLa cells treated with (**a**) Rh-labeled pDNA-containing complexes or (**b**) Rh-PE-containing complexes for 24 h with or without 1 min-NIR laser irradiation at 1 h after complex apply (*n* = 2). Statistical analyses were done using a two-tailed Student’s *t*-test. ns: not significant.

**Figure 5:**
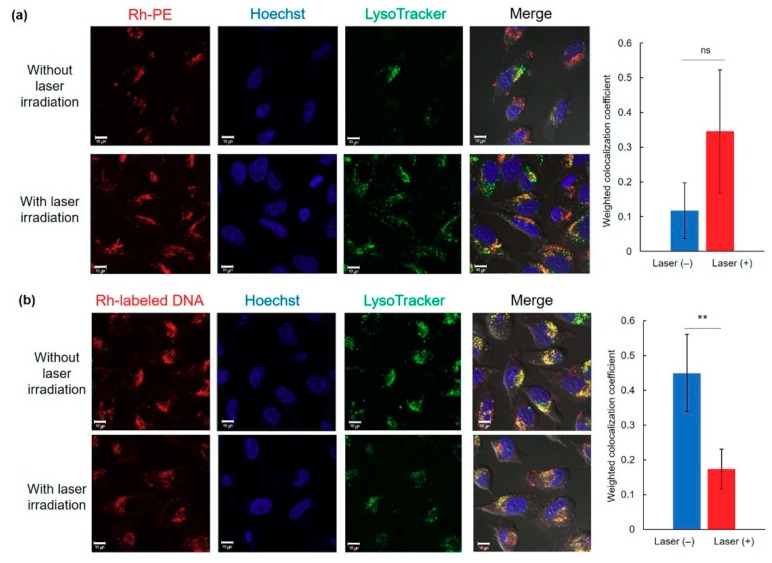
Intracellular distribution of Rh-PE-labeled complexes (**a**) or Rh-labeled pDNA (**b**) at 24 h with or without 1 min-laser irradiation at 1 h after sample apply. Cell nucleus and endo/lysosomes were also stained with Hoechst and LysoTracker Green, respectively. Scale bars represents 10 µm. Weighted colocalization coefficients of Rh fluorescence overlapped with LysoTracker fluorescence from each CLSM image were also shown (*n* = 5–7). Statistical analyses were done using a two-tailed Student’s *t*-test. ** *p* < 0.01. ns: not significant.

**Figure 6:**
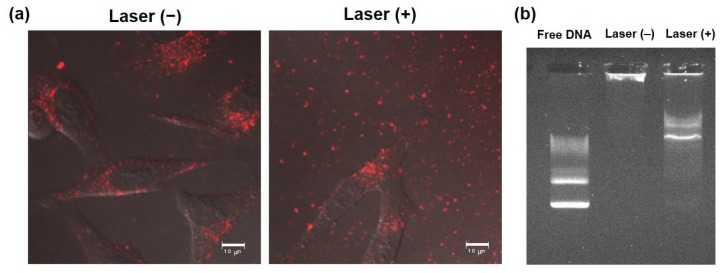
Effect of NIR laser irradiation on interaction with cells and DNA release from complexes. (**a**) Confocal laser scanning microscopic images of HeLa cells treated with Rh-labeled pDNA-containing complexes for 1 h and with or without subsequent 1 min-laser irradiation. Cells were observed at 30 min after laser irradiation without washing. Scale bars represent 10 µm. (**b**) DNA release under NIR laser irradiation. Complexes with or without 1 min-laser irradiation were electrophoresed and fluorescent bands derived from DNA were observed.

To elucidate the transfection activity improvement mechanism under laser irradiation condition II, cellular association and intracellular distribution of hybrid vector and pDNA were investigated using fluorescence-labeled complexes. Laser irradiation at 1 h did not show significant increase of cellular association of Rh-labeled pDNA-containing complexes (Figure 4a) and Rh-PE-labeled complexes (Figure 4b) at 24 h. Therefore, the difference in intracellular behavior of complexes might lead to improvement of transfection activity. First, intracellular distribution of Rh-PE-labeled complexes after 24 h incubation was observed using CLSM (Figure 5a). Irrespective of the laser irradiation, Rh-PE-labeled complexes were internalized into the cells (Figure 5a). Colocalization analysis between Rh-PE-labeled complexes and endo/lysosomes also did not show significant difference irrespective with laser irradiation (Figure 5a). Next, intracellular distribution of pDNA was analyzed using Rh-labeled pDNA (Figure 5b). Many punctate fluorescence dots were observed in both transfected cells with or without laser irradiation (Figure 5b). Most pDNA fluorescence dots were overlapped with endo/lysosome fluorescence for the cells without laser irradiation, whereas pDNA fluorescence dots were located at different sites with endo/lysosome fluorescence in the case where laser irradiation at 1 h was applied. These observations were confirmed further by colocalization analysis of Rh-DNA and endo/lyosome (Figure 5b), suggesting that endosome escape of pDNA was promoted by 1-min laser irradiation at 1 h. Figure 5 shows the location of complexes after 24 h, whereas earlier cellular association process might cause such differences in intracellular distribution of pDNA. Therefore, pDNA location immediately after laser irradiation at 1 h after complex application was observed (Figure 6a). Compared with no laser irradiation group, laser irradiation apparently promoted the absorption of pDNA-derived fluorescence onto a glass substrate and the internalization of pDNA into the cells. Laser irradiation might cause not only the increase of adsorption to the cells through dehydration of EDEG moieties but also the release of DNA from the complexes through heating surrounding of AuNR. Actually, DNA-derived fluorescence bands were observed at higher molecular size position compared with free pDNA after laser irradiation (Figure 6b), suggesting that laser irradiation promoted the release of pDNA from the complexes.

## 4. Discussion

Precise control of transfection activity of gene vectors by external stimuli is expected to be promising as a site-specific or cell-specific transfection system for gene therapy without adverse events. Among external stimuli, light is applicable to spatiotemporal control of photoresponsiveness [30]. For this study, we prepared light-responsive and temperature-responsive nanohybrids for selective enhancement of gene expression at light-irradiated sites or cells. As a light-responsive material, AuNR was used because AuNR shows strong absorption at the NIR region, which has high permeability into the body and which also has heat generation properties under NIR light absorption [8,9,10]. As a temperature-responsive material, oligo (ethylene glycol)-modified dendron-bearing lipid (EDEG-DL) was used for the control of interaction with cells or endosomal membrane in response to temperature changes [25]. After mixing with carboxy surface AuNRs and DL suspension that contains EDEG-DL and amino-terminated DL, submicrometer-sized ellipsoidal particles containing multiple AuNRs (hybrid vector) were obtained (Figure 2c,f and Figure S1, and Table 1). Importantly, hybrid vector retains light absorption property at NIR region like parental MUA-AuNR (Figure 2h), indicating that AuNRs did not form irreversible aggregates even after encapsulation into DL assemblies. Furthermore, the heat generation property of hybrid vector was quite high compared to those of MUA-AuNRs (Figure 2i). This high heat generation suggests that the proximity of AuNRs inside of hybrid vectors promotes light-to-heat conversion efficacy [31] or plasmonic hot spots between assembled AuNRs in hybrid vectors might enhance the heat generation property [32,33]. In addition, considering the aggregation of MUA-AuNRs takes place at neutral pH [30], suppression of aggregation through AuNR coverage by DL molecules might also contribute to retain the heat generation behavior in a physiological condition.

Hybrid vector also has positive zeta potential derived from DL, which is useful for complex formation with anionic molecules. Actually, hybrid vector formed complexes with pDNA (Figure S2). Considering the increase of AuNRs per particle, as shown in TEM images (Figure 2g and Figure S1), multiple hybrid vectors were cross-linked by pDNA via electrostatic interaction. From TEM images for complex (Figure S1), particles with one or two AuNRs were observed. At L/P = 48, the excess hybrid vectors that did not associate to complex formation with pDNA might be observed as such particles with one or two AuNRs in the TEM images. Obtained complexes showed markedly enhanced photothermal property and decreased transition temperature compared to the parent DL suspension (Figure 2j). Complex formation with pDNA and hybrid vector might produce hydrophobic domains inside of the complexes, which promotes the dehydration of EDEG moieties at lower temperatures. Complexes with pDNA induced changes in transmittance and particle sized at more than 40 °C, which is a suitable temperature for photothermal application without adverse events by excess heating.

Short-time NIR laser irradiation applied soon after sample apply was effective for improving the transfection activity of the complexes (Figure 3). Two-minute laser irradiation to complexes caused a 12 °C increase in the bulk temperature of the culture medium (Figure 2i), which suggests that a quite high temperature was created at the local area surrounding AuNRs. Actually, laser irradiation for more than 2 min might cause cell activity dysfunction. Results show that laser irradiation to the cells at late timing (24 h) did not affect transfection activity (Figure 3). After 24 h incubation, most complexes were internalized into cells and were located in endo/lysosomes (Figure 5). At acidic pH, transition temperatures of EDEG-DL increased compared with those of neutral pH [25]. Therefore, 1 min laser irradiation might be insufficient to induce dehydration of EDEG moieties and endosomal escape via hydrophobic interaction. At 1 h after sample application, most complexes still exist in the culture medium. When NIR laser light was irradiated to the medium, AuNRs generate heat to induce dehydration of EDEG moieties. After dehydration of EDEG moieties, surface properties of the complexes change from hydrophilic to hydrophobic, thereby promoting the absorption and internalization of the complexes into cells via hydrophobic interaction (Figure 6a). After laser irradiation to the complexes, the bands were observed apparently higher molecular size position compared with free DNA (Figure 6b). This suggests that released DNA loosely bound to the components of hybrid vector, especially cationic dendron lipids even after laser irradiation, resulting in the increase of apparent molecular size of released DNA compared with free DNA. Such release of DNA or loose complexes might enhance internalization of pDNA into cytoplasm after laser irradiation (Figure 4).

Nakatsuji et al. investigated the effect of surface chemistry of AuNR on cytosolic gene delivery [34]. Furthermore, they found that short-time (10–60 s) NIR laser irradiation at 24 h after sample applying promoted the cytosolic release of gene, resulting in increased transfection efficiency [34]. Thus, heat generation under NIR light irradiation is useful to control the intracellular distribution of gene. NIR light-activatable other transfection systems such as indocyanine green-introduced layer-by-layer nanoparticles [35], poly(ethylene imine)-modified carbon nanotubes [36], and gold nanostar-incorporated liposomes [37] could also control or promote the transfection by enhanced endosomal escape of gene or liberation of gene from the complexes using light irradiation. Compared with these reports, our transfection system can control cellular internalization and endosomal escape process using dually light- and temperature-responsive moieties, AuNR and EDEG-DL, respectively (Figure 5 and Figure 6), which would provide more precisely controlled gene transfection using NIR light. In terms of transfection activity, GFP-positive cells have not been high so much compared with other literatures or parental dendron lipid-based transfection systems [19,38], probably because hydrophilic EDEG-coated surface of complexes suppressed the cellular association of complexes during sample incubation with cells, whereas surface hydrophobized complexes by 1 min-laser irradiation effectively internalized to the cells. Further improvements to promote the cellular association of complexes such as introduction of ligand molecules exposed after laser irradiation are required towards future application to in vitro/in vivo controlled gene transfection systems.

## 5. Conclusions

For this study, a AuNR-based hybrid vector covered with thermosensitive EDEG−DL and amino group-terminated DL was designed for light-controlled transfection. The hybrid vector formed submicrometer-sized complexes with pDNA. Complexes generated heat under NIR laser irradiation and formed aggregates at high temperatures. Transfection activity of the complexes was improved considerably by short NIR laser irradiation applied soon after sample apply, which might result from enhanced internalization of the complexes via hydrophobic interaction and release of pDNA. Results demonstrate that hybrid vector-pDNA complex is promising as a nanoplatform for a light-activatable transfection system for spatiotemporal control of gene delivery. Towards clinical application of such controlled transfection system, further evaluations such as feasibility to other cell lines/primary cells or biodistribution of complexes after intravenous injection and improvement of transfection selectivity through masked targeting molecule introduction are required.

## Figures and Tables

**Table 1:** Particle sizes and zeta potentials of MUA-AuNR, DL suspension, hybrid vector and complex (L/P = 48).

Sample	Temperature (°C)	Particle Size (nm)	Zeta Potential (mV)
MUA-AuNR	25	36 ± 1	−23.5 ± 1.7
DL suspension	25	724 ± 73	9.19 ± 0.2
	50	2565 ± 248	–
Hybrid vector	25	135 ± 12	4.19 ± 0.4
	50	297 ± 19	–
Complex	25	185 ± 50	3.66 ± 0.8
	50	300 ± 21	–

## Supplementary Materials

The following are available online at https://www.mdpi.com/1999-4923/12/3/239/s1, Scheme S1: Synthetic route for EDEG-DL, Figure S1: TEM images for AuNR, hybrid vector and complex, Figure S2: Agarose gel electrophoretic analysis of complex formation between pDNA and hybrid vector, Figure S3: Optimization of lipid/DNA ratio on transfection activity, Figure S4: Optimization of NIR laser irradiation time on transfection activity, Figure S5: Histograms for GFP expression on the cells.
