# Supplementary material for: Light-Activatable Transfection System Using Hybrid Vectors Composed of Thermosensitive Dendron Lipids and Gold Nanorods
Source: Pharmaceutics. 2020 Mar 7;12(3):239. doi: 10.3390/pharmaceutics12030239 (PMC7150951; doi:10.3390/pharmaceutics12030239)
Supplement: Supplementary file 1 [file pharmaceutics-12-00239-s001.pdf]

pharmaceutics

Supplementary Materials: Light-Activatable Transfection System Using Hybrid Vectors Composed of Thermosensitive Dendron Lipids and Gold Nanorods

Takuya Hashimoto, Tomoya Hirata, Eiji Yuba, Atsushi Harada and Kenji Kono

Synthesis of temperature-sensitive dendron lipid (EDEG-DL) (Scheme S1)

Polyamidoamine generation-2 dendron-bearing lipid having two octadecyl chains (DL–S-G2.0) were synthesized according to previous report (Takahashi, T., Kojima, C., Harada, A., and Kono, K. (**2007**) Alkyl chain moieties of polyamidoamine dendron-bearing lipids influence their function as a nonviral gene vector. *Bioconjugate Chem.* **18**, 1349– 1354). To a solution of DL–S-G2.0 (200 mg, 151.5 μmol) in dichloromethane (10 mL), the solution of EDEG-4-nitrophenyl chloroformate (544 mg, 1818 μmol) in dichloromethane (3 mL) was added dropwise under nitrogen. After 5 days at room temperature, solvent was evaporated under vacuum, and the residue was chromatographed on silica gel using chloroform–methanol (9/1 subsequently 8/2, v/v) as an eluent. The yield was 120 mg, 40%. ^{1}H NMR (CDCl$_3$, 400 MHz): δ 0.87 (m, C*H*$_3$(CH$_2$)$_{15}$-), δ 1.20 (t, -OCH$_2$C*H*$_3$), δ 1.25 (t, CH$_3$(C*H*$_2$)$_{15}$-), δ 1.44 (m, -C*H*$_2$CH$_2$N-), δ 2.36 (m, -NCH$_2$C*H*$_2$CO-), δ 2.52 (t, CH$_3$(C*H*$_2$)$_{16}$C*H*$_2$N-), δ 2.71 (m, -NHCH$_2$C*H*$_2$N-, -NC*H*$_2$CH$_2$CO-), δ 3.27 (t, -NHCH$_2$C*H*$_2$NH-), δ 3.33 (t, -NHC*H*$_2$CH$_2$N-), δ 3.51 (m, -OC*H*$_2$CH$_3$), δ 3.57 (t, -NHC*H*$_2$CH$_2$NH-), δ 3.62 (t, -O(C*H*$_2$)$_2$OCH$_2$CH$_3$), δ 3.67 (t, -COOCH$_2$C*H*$_2$O-), δ 4.19 (t, -COOC*H*$_2$CH$_2$O-), δ 6.10 and 7.50 (m, -N*H*-).

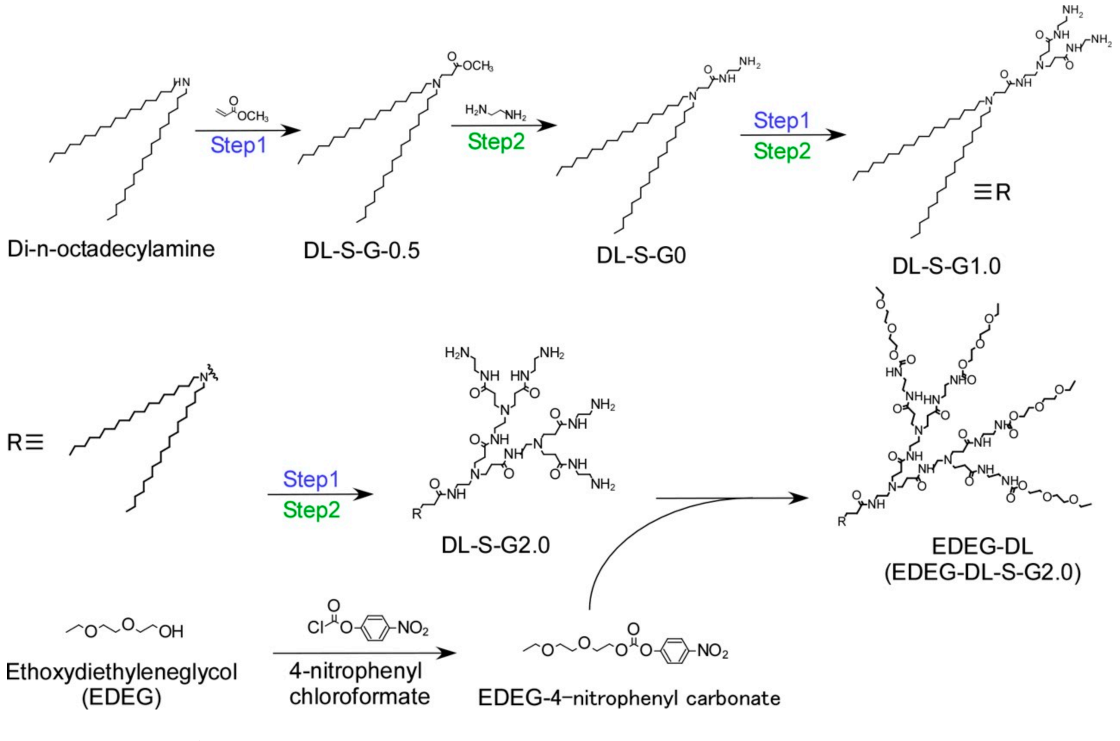

Scheme S1. Synthetic route of thermosensitive dendron lipid (EDEG-DL).

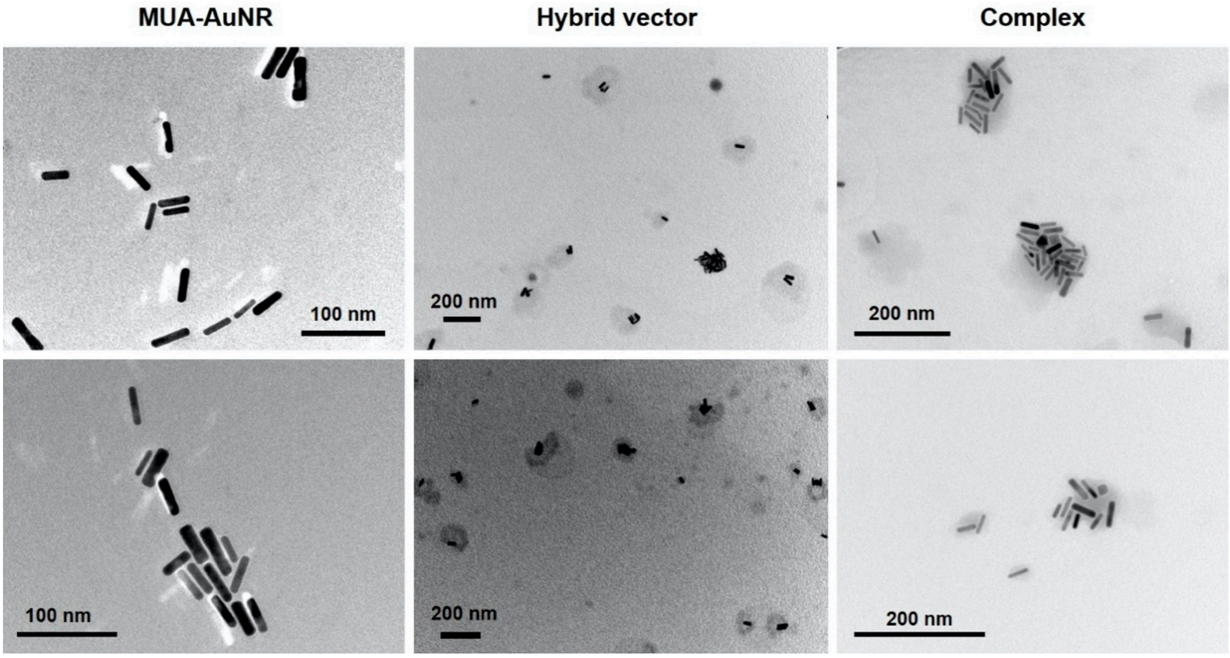

Figure S1. Transmission electron microscopic (TEM) images for MUA-AuNR, hybrid vector, and complex at low magnification.

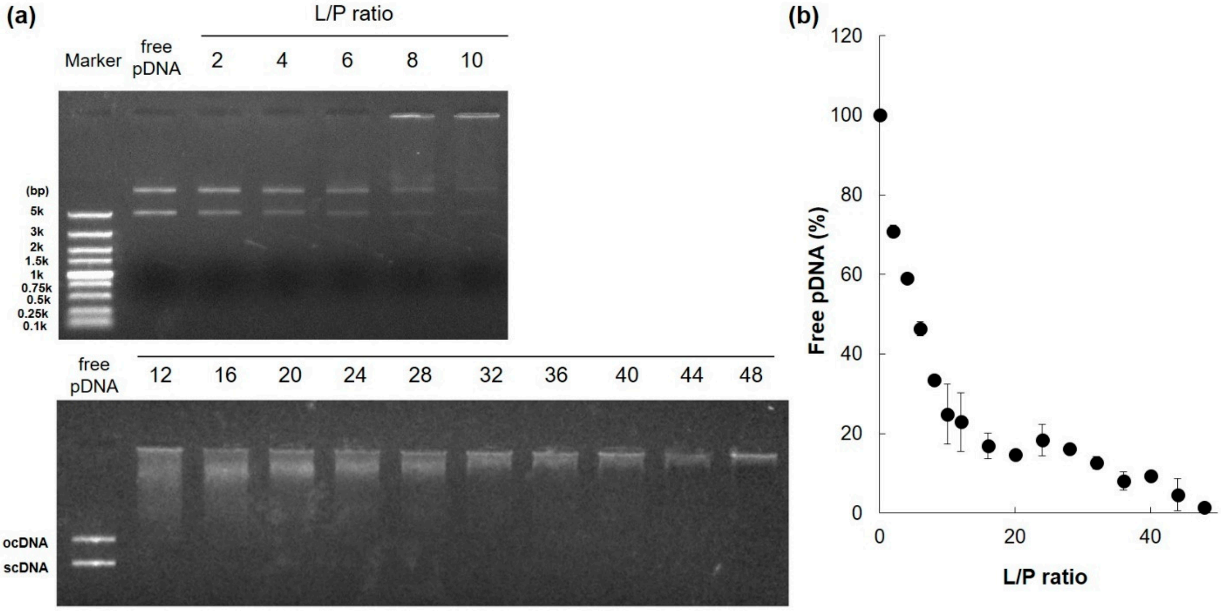

Figure S2. (**a**) Representative agarose electrophoresis images for pDNA-hybrid vector complexes. ocDNA: open circular DNA, scDNA; supercoiled DNA. (**b**) Percentage of residual pDNA as a function of L/P ratio (*n* = 3). "L" represents lipid component in DL suspension (EDEG-DL/DL = 9/1, mol/mol).

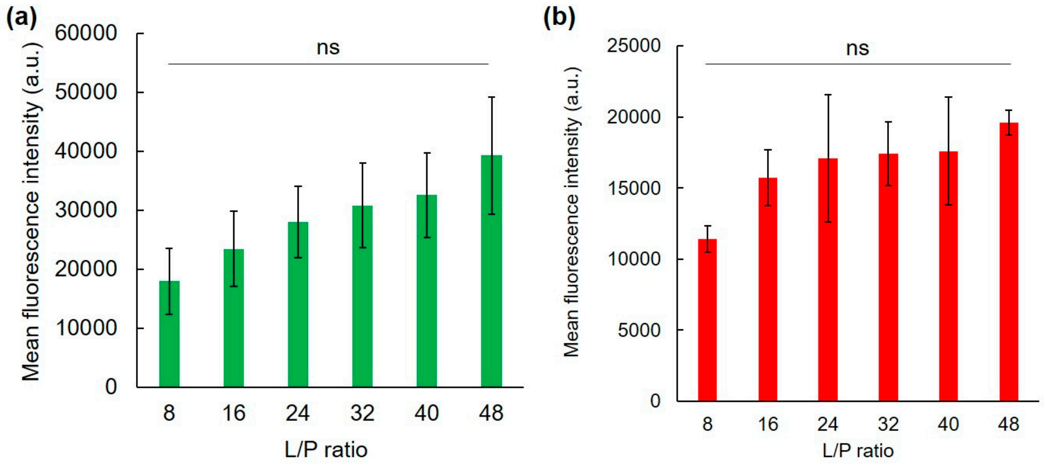

Figure S3. Effect of L/P ratio on transfection activity (**a**) and cellular association (**b**) of pDNA-hybrid vector complexes (n = 2). Statistical analyses were done using analysis of variance (ANOVA) with Tukey's test. ns: not significant.

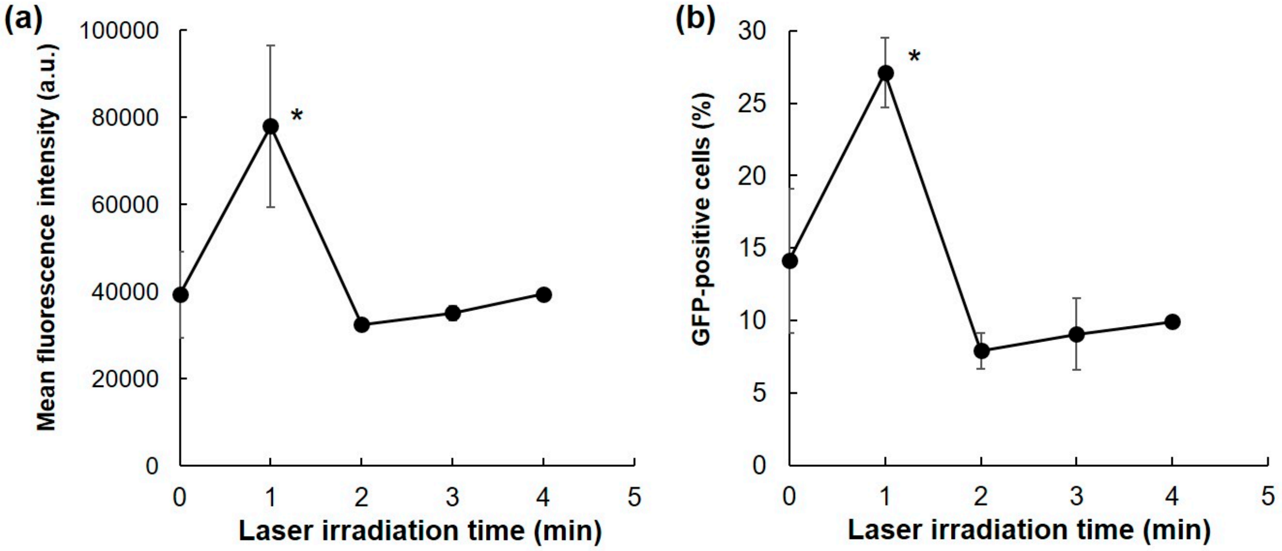

Figure S4. Effect of NIR laser irradiation time on transfection activity of pDNA-hybrid vector complexes (L/P = 48). (**a**) GFP expression levels and (**b**) percentage of GFP-positive cells were measured using a flow cytometer. After 1 h-incubation with EGFP-encoding pDNA-loaded complexes, NIR laser (3.5 W/cm^2) was irradiated to HeLa cells for 0-5 minutes. Cells were washed with PBS at 24 h after sample apply, and then incubated with culture medium for additional 24 h before flow cytometric analysis (n = 2-4). Statistical analyses were done using analysis of variance (ANOVA) with Tukey's test. *$P < 0.05$ compared with other groups.

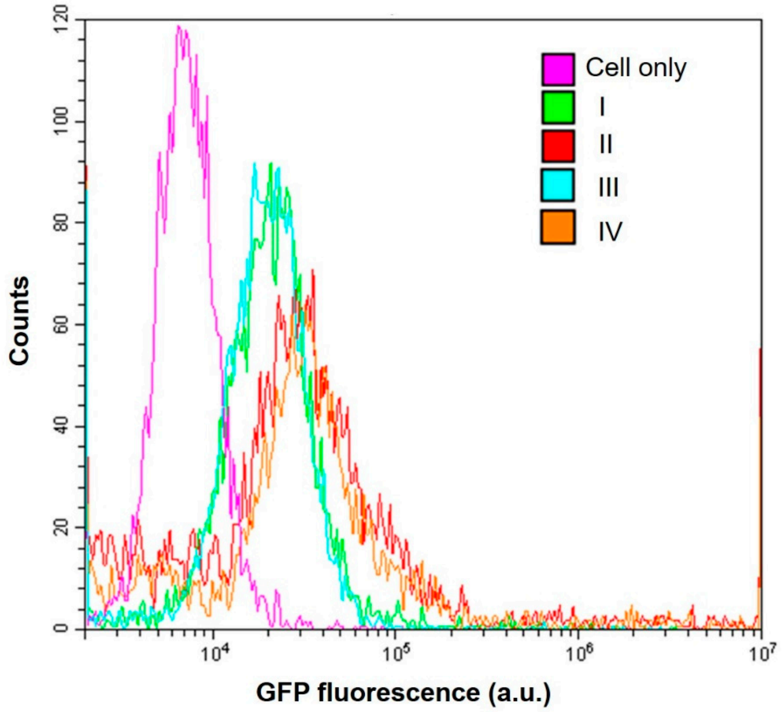

Figure S5. Representative histograms of GFP expression levels on HeLa cells in Figure 3c.
